# Effects of pH on the microarchitecture of carbonate apatite granules fabricated through a dissolution–precipitation reaction

**DOI:** 10.3389/fbioe.2024.1396275

**Published:** 2024-06-06

**Authors:** Zhibin Wang, Masaya Shimabukuro, Ryo Kishida, Taishi Yokoi, Masakazu Kawashita

**Affiliations:** ^1^ Graduate School of Medical and Dental Sciences, Tokyo Medical and Dental University, Tokyo, Japan; ^2^ Institute of Biomaterials and Bioengineering, Tokyo Medical and Dental University, Tokyo, Japan; ^3^ Faculty of Dental Science, Kyushu University, Fukuoka, Japan

**Keywords:** apatite, carbonate apatite, dissolution–precipitation reaction, composition, architecture

## Abstract

Both the composition and architecture of artificial bone govern bone regeneration. Herein, carbonate apatite (CAp), which has a similar mineral composition to bone, was prepared by immersing calcium carbonate (CaCO_3_) in a phosphate solution with varying acidification levels (pH 6.0) to pH 8.9, to reveal the influence of pH on the composition and architecture of the resultant CAp granules. The composition, crystal morphology, and architecture of resultant CAp granules was well-characterized by X-ray diffraction, scanning electron microscopy, mercury intrusion porosimetry and so on. Consequently, the rate of compositional transformation from CaCO_3_ to CAp was much higher at pH 6.0 and pH 7.0 than pH 8.0 and pH 8.9. The pH of the phosphate solution did not affect the macroarchitecture of the resultant CAp granules. In contrast, the composition, crystal morphology, microarchitecture, and degradation behavior of the resultant CAp granules were affected by pH of the phosphate solution. In particular, the open-pore distributions and volumes of the CAp granules prepared at pH 6.0–8.9 were changed to reflect the microarchitecture of the samples. Therefore, this study revealed that the pH-controlled elution precipitation reaction is useful for controlling the composition, crystal morphology, microarchitecture, and degradation behavior of the resultant CAp, while preserving its macroarchitecture. Our findings provide fundamental insights into the design of artificial bones for bone regeneration.

## 1 Introduction

The demand for bone regeneration is increasing in the medical field owing to the aging global population (Martyniak et al., 2021; Vann, 2023). Although autogenous bone grafting is currently the gold standard treatment for bone regeneration, it is limited by supply constraints, high morbidity rates, donor-site pain, and potential graft resorption (Betz, 2002; Oryan et al., 2014; de Azambuja Carvalho et al., 2019). For overcoming these limitations, calcium phosphate artificial bones are expected to be an effective alternative to autogenous bone grafting (Ho-Shui-Ling et al., 2018; Zhou et al., 2021). Natural bone mostly consists of apatite cross-linked with type I collagen (Glimcher, 2006), and apatite contains a small amount of carbonate depending on its source, species, and age (Landi et al., 2003; Boskey and Coleman, 2010). Thus, as a significant component of artificial bones, carbonate apatite [CAp: Ca_10-a_(PO_4_)_6-b_(CO_3_)_c_] is of particular interest given its inorganic components.

The chemical composition and architecture of artificial bone are important factors directly related to its bone regeneration capability (Shao et al., 2017; Latimer et al., 2021; Wu et al., 2021). From a macroscopic perspective, the presence of pores >100 μm in diameter allows the migration of cells and tissues from the surrounding host tissues, promoting osteogenesis and angiogenesis in artificial bone (Hayashi et al., 2020; Hayashi and Ishikawa, 2021). In contrast, from a microscopic perspective, pores smaller than a few hundred nanometers accelerate the biodegradation of artificial bone by promoting osteoclastogenesis (Hayashi and Ishikawa, 2020). Thus, artificial bones should be precisely designed from micro- to macro-architecture. CAp is currently prepared by chemical conversion through a dissolution–precipitation reaction using a precursor (Ishikawa and Hayashi, 2021). A dissolution–precipitation reaction, which utilizes reactions in the liquid phase, can convert the precursor calcium carbonate (CaCO_3_) to CAp, while maintaining the macroarchitecture (Ishikawa and Hayashi, 2021). In a dissolution–precipitation reaction, because the reaction system is supersaturated with respect to CAp, CAp precipitates immediately when CaCO_3_ dissolves. Generally, the solubility of CaCO_3_ is affected by the pH of the solution (Coto et al., 2012). Therefore, it is expected that the dissolution of CaCO_3_ and the accompanying precipitation of CAp are significantly affected by the pH of the reaction system. In other words, pH-controlled dissolution–precipitation reactions are useful for controlling the microarchitecture of CAp prepared from CaCO_3_, and may provide important knowledge in the development of superior bioabsorbable artificial bones. However, no reports of pH-controlled dissolution–precipitation reactions exist on fabrication of CAp.

In this study, CAp granules were prepared from CaCO_3_ granules through a dissolution–precipitation reaction under acidic, neutral, and alkaline conditions. To clarify the effect of pH, the composition and architecture of the resulting CAp were characterized.

## 2 Materials and methods

### 2.1 Sample preparation

As described in our previous study (Shimabukuro et al., 2022), the spherical aggregates of CaCO_3_ crystals (SCS-M5, Sakai Chemical Industry Co., Ltd., Osaka, Japan) were placed in a stainless-steel mold and pressed uniaxially using an oil pressure machine at 150 MPa. They were heated to 350°C at a rate of 5°C·min^−1^ and held at this temperature for 24 h. After heating, the samples were crushed and sieved to obtain 600–1,000 μm CaCO_3_ granules. Finally, these granules were immersed in a 1 mol L^−1^ sodium hydrogen phosphate (Na_2_HPO_4_; Fujifilm Wako Pure Chemical, Osaka, Japan) solution adjusted to pH 6, 7, 8, and 8.9 using a phosphoric acid (H_3_PO_4_; Fujifilm Wako Pure Chemical), at 80°C for 5–14 days. This reaction system was prepared at 0.05 molar ratio of CaCO_3_ and Na2HPO4 (CaCO_3_/Na_2_HPO_4_). After immersion, the obtained CAp granules were washed for 30 min by immersion in distilled water at 80°C. The CAp granules prepared at pH 6.0, 7.0, 8.0, and 8.9 were designated 6-CA, 7-CA, 8-CA, and 8.9-CA, respectively.

### 2.2 Sample characterization

The crystalline phases of 6-CA, 7-CA, 8-CA, and 8.9-CA were characterized by X-ray diffraction (XRD; MiniFlex600; Rigaku Corp., Tokyo, Japan) employing CuKα radiation. Diffraction patterns were recorded from 25° to 40° at a step size of 0.02°. The apatite lattice spacings along the a- and c-axes were determined from the XRD patterns of the samples using sodium fluoride (Fujifilm Wako Pure Chemical) as an internal reference. The carbonate content of the samples was measured using elemental carbon-hydrogen-nitrogen (CHN) analysis (MT-6, Yanako Analytical Instruments, Kyoto, Japan). Before CHN analysis, all samples were heated at 250°C for 3 h to remove carbon contamination. Fourier-transform infrared (FT-IR) spectroscopy (FT/IR-6200; JASCO, Tokyo, Japan) was used to analyze the chemical structures of the samples. As described elsewhere (Madupalli et al., 2017; Shimabukuro et al., 2022), the A-site and B-site carbonates in AB-type CAp were calculated from the FT-IR spectra and CHN results. Elemental analysis for measuring the calcium and phosphorous contents was performed by inductively coupled plasma-atomic emission spectrometry (ICP-AES; ICPE-9800; Shimadzu Corp., Kyoto, Japan). The macro- and microarchitectures of 6-CA, 7-CA, 8-CA, and 8.9-CA were examined using an S9D microscope (Leica, Wetzlar, Germany) equipped with a Digital Sight 1000 camera (Nikon, Tokyo, Japan) and a field-emission scanning electron microscope (JSM-7900F; JEOL, Tokyo, Japan). The size distribution and volume of the open pores in the samples were determined using mercury intrusion porosimetry (MIP; AutoPore 9420, Shimadzu Corp., Kyoto, Japan). The samples were immersed in a physiological saline solution (0.9% NaCl) at 37°C during 1, 3, and 5 days. The Ca and phosphate ions released from each sample were determined by ICP-AES. All values obtained by elemental analysis are presented as means ± standard deviation, and commercial statistical software Kaleida Graph software (version 4.1.1; Synergy Software, Eden Prairie, MN) was used for statistical analysis. One-way analysis of variance was used followed by multiple comparisons with the Student–Newman–Keuls method to assess the data, and *p* < 0.05 was considered to indicate statistical significance.

## 3 Results and discussion

**FIGURE 1:**
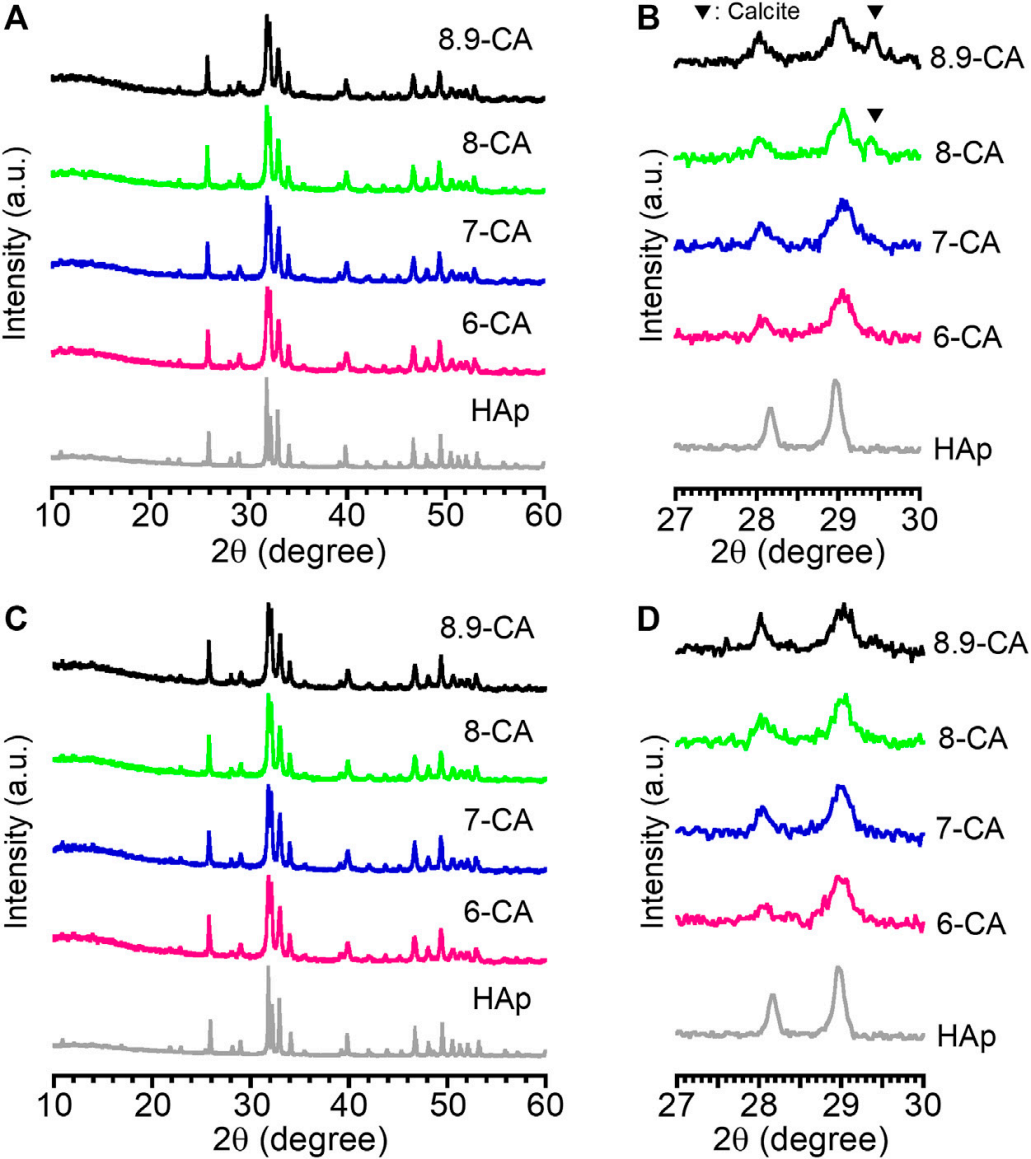
X-ray diffraction patterns at 10°–60° **(A, C)** and those at 27°–30° **(B, D)** of CAp granules prepared after 5 days **(A, B)** and 14 days **(C, D)** of immersion in phosphate solutions at pH 6.0 (6-CA), 7.0 (7-CA), 8.0 (8-CA), and 8.9 (8.9-CA).

After 5 days of immersion in phosphate solutions with different pH values, XRD peaks attributed to apatite were observed in all samples, and XRD peaks attributed to CaCO_3_ were observed only for 8-CA and 8.9-CA (Figures 1A, B). Moreover, there were no CaCO_3_ peaks in the XRD patterns of 6-CA and 7-CA. After 14 days of immersion, no peaks attributed to CaCO_3_ were observed in any of the samples, regardless of the pH of the phosphate solution (Figures 1C, D). In other words, CaCO_3_ was completely converted to apatite under all pH conditions after 14 days of immersion. Therefore, in this study, we characterized both the composition and architecture of the samples after 14 days of immersion.

**FIGURE 2:**
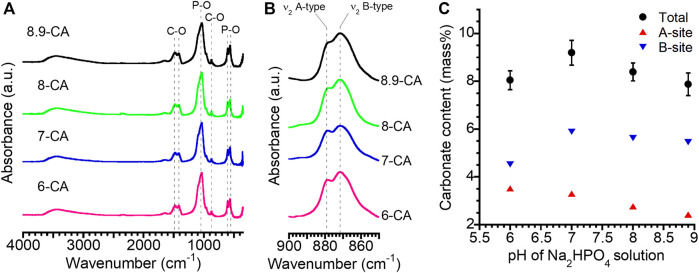
Fourier-transform infrared spectra at 4,000 to 350 cm^−1^ **(A)** and 900 to 850 cm^−1^ **(B)** obtained from 6-CA, 7-CA, 8-CA, and 8.9-CA. Changes in the carbonate content with the pH of the Na_2_HPO_4_ solution **(C)**. These results were obtained from the samples after 14 days of immersion in an Na_2_HPO_4_ solution at pH 6.0 to 8.9.

FT-IR absorption bands due to phosphate (1,154–963 and 614–519 cm^−1^) and carbonate (1,539–1,364 cm^−1^: ν_3_ region, 890–860 cm^−1^: ν_2_ region) groups were observed regardless of the pH of the Na_2_HPO_4_ solution (Figures 2A, B). No absorption bands ascribed to hydroxyl groups were detected in any of the samples. This indicated that the samples prepared from phosphate solution at pH 6.0 to 8.9 were mainly composed of apatite, in which the OH^–^ and PO_4_^3–^ sites were completely or partially substituted by CO_3_^2–^. Thus, the AB-type CAp was fabricated from CaCO_3_ via a dissolution–precipitation reaction (Tan et al., 2023; Tan et al., 2024). The total carbonate contents in 6-CA, 7-CA, 8-CA, and 8.9-CA were 8.0, 9.2, 8.4, and 7.9 mass%, respectively (Figure 2C). Moreover, Ca/P ratios of 6-CA, 7-CA, 8-CA, and 8.9-CA were 1.78, 1.81, 1.94 and 1.92, respectively (Table 1). These results revealed that AB-type CAp with different compositions was fabricated when CaCO_3_ was immersed in a phosphate solution at pH 6.0–8.9. This compositional transformation from CaCO_3_ to CAp was caused by both the dissolution of CaCO_3_ (Eq. 1) and the precipitation of CAp (Eq. 2), that is, the dissolution–precipitation reaction.

$${\text{CaCO}}_{3}\to {\text{Ca}}^{2+}+{\text{CO}}_{3}^{2-}$$ (1)

$${\text{Ca}}^{2+}+{\text{PO}}_{4}^{3-}+{\text{CO}}_{3}^{2-}\to {\text{Ca}}_{10-a}{\left({\text{PO}}_{4}\right)}_{6-b}{\left({\text{CO}}_{3}\right)}_{c}$$ (2)

**TABLE 1:** Calcium to phosphorous (Ca/P) ratio determined by ICP-AES.

Sample	Ca/P ratio
6-CA	1.78 ± 0.06
7-CA	1.81 ± 0.01
8-CA	1.94 ± 0.05
8.9-CA	1.92 ± 0.01

The rate of the compositional transformation from CaCO_3_ to CAp was affected by the pH of the phosphate solution. No CaCO_3_ peaks were detected in the XRD patterns of the samples after 5 days of immersion in phosphate solutions at pH 6.0 and 7.0, suggesting accelerated compositional transformation from CaCO_3_ to CAp under relatively acidic conditions. This indicated that the acidic conditions in the phosphate solutions promoted the dissolution of CaCO_3_, resulting in the rapid precipitation of CAp. Bingel et al. (2015) studied the influence of dissolution medium pH on ion release and apatite formation in Bioglass^®^ 45S5. They concluded that ion release occurred significantly faster at low pH values, resulting in significantly faster apatite formation. Since the compositional transformation from CaCO_3_ to CAp was accelerated at pH 6.0 and 7.0 compared with pH 8.0 and 8.9, our results were consistent with theirs. Thus, the pH of the phosphate solution governs the compositional transformation of CaCO_3_ to CAp during the dissolution and precipitation reactions.

Moreover, the carbonate content at site A tended to decrease as the pH increased. In contrast, it was revealed that the carbonate content at the B site increased between pH 6.0 and pH 7.0, and then remained almost constant at pH 7.0 to pH 8.9. The a-axis lattice parameters calculated from the XRD patterns of 6-CA, 7-CA, 8-CA, and 8.9-CA were 9.404, 9.399, 9.402, and 9.404 Å, respectively. The c-axis lattice parameters of these samples were 6.910, 6.912, 6.914, and 6.918 Å, respectively. Thus, the pH of the phosphate solution slightly affected the a- and c-axis lattice spacings of apatite. In general, the a- and c-axis lattice parameters of hydroxyapatite are 9.423 and 6.889 Å, respectively (Shimabukuro et al., 2022). The a-axis spacing of the samples was shorter and the c-axis spacing of the samples was longer than that of hydroxyapatite. Some researchers have reported the influence of carbonate substitution in apatite on the lattice spacing of the a- and c-axes. In the substitution of carbonate groups at the A-site, two hydroxyl groups are replaced by one, and the substituted carbonates are oriented parallel to the a-plane; therefore, the c-axis expands as the number of carbonate groups at the A-site decreases (Shimabukuro et al., 2022). Furthermore, in the substitution of carbonate groups at the B-site, the changes in apatite lattice spacing are affected by the substitution from larger tetrahedral phosphate to smaller trigonal planar carbonate. Thus, the c-axis lattice spacing increases and the a-axis lattice spacing decreases (Wingender et al., 2021). Therefore, our results showing changes in the a- and c-axis lattice spacings by the substitution of carbonate groups were consistent with those of previous studies. In particular, our results revealed that the c-axis lattice spacing increased as the carbonate content of the A site decreased, because the carbonate at the B site was similar among 7-CA, 8-CA, and 8.9-CA. This suggested that in the substitution of carbonate groups with AB-type CAp, substitution at the A-site particularly contributed to the decrease in the c-axis lattice spacing. Thus, the pH of the phosphate solution can control the lattice spacing of CAp fabricated from CaCO_3_ through a dissolution–precipitation reaction.

**FIGURE 3:**
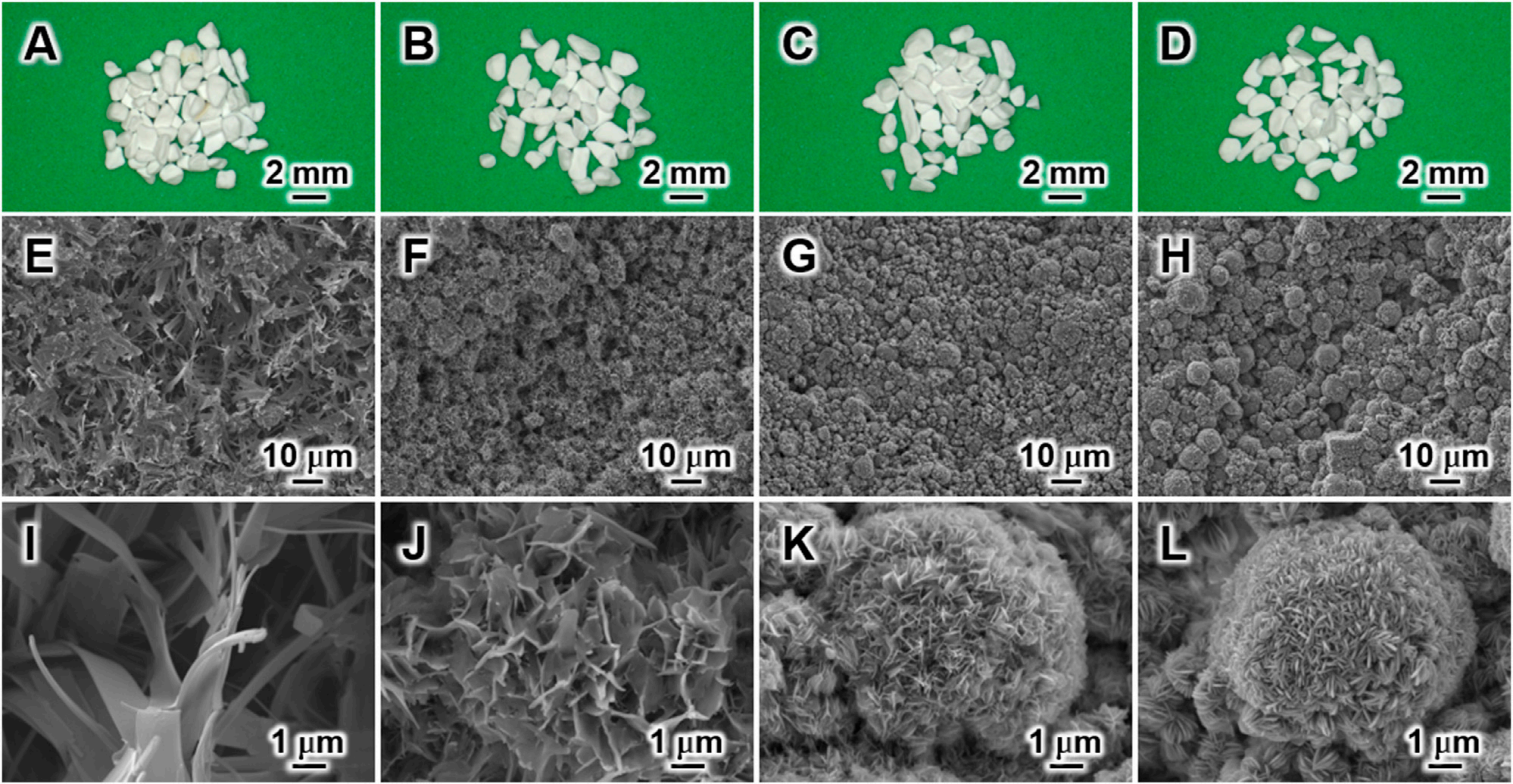
Stereoscopic microscopy **(A–D)** and field-emission scanning electron microscopy **(E–L)** images of 6-CA **(A, E, I)**, 7-CA **(B, F, J)**, 8-CA **(C, G, K)**, and 8.9-CA **(D, H, L)**.

Stereoscopic microscopy images revealed that 6-CA, 7-CA, 8-CA, and 8.9-CA retained their granular shapes even after 14 days of immersion under all pH conditions (Figures 3A–D). This indicates that the dissolution–precipitation reaction at pH 6.0 to 8.9 led to the compositional conversion from CaCO_3_ to apatite without macro-structural alterations. In contrast, there were differences in the microarchitecture of the samples (Figures 3E–L). Spherical aggregates were observed in 7-CA, 8-CA, and 8.9-CA (Figures 3F–H), whereas plate-like aggregates were observed only in 6-CA (Figure 3E). Furthermore, high-magnification images revealed that scale-like crystals were detected in 8-CA and 8.9-CA, and these crystals were similar (Figures 3K, L). Thus, the 8.9-CA and 8-CA granules were formed by the interconnection of spherical aggregates comprising scale-like crystals. In contrast, plate-like and branch-like crystals were observed for 7-CA and 6-CA (Figures 3I, J). Thus, 7-CA granules were constructed by interconnecting spherical aggregates comprising plate-like crystals, and 6-CA granules were constructed by interconnecting plate aggregates comprising branch-like crystals. Thus, the pH-controlled dissolution–precipitation reaction resulted in the formation of CAp granules with the remaining macrostructure and microstructural alterations.

**FIGURE 4:**
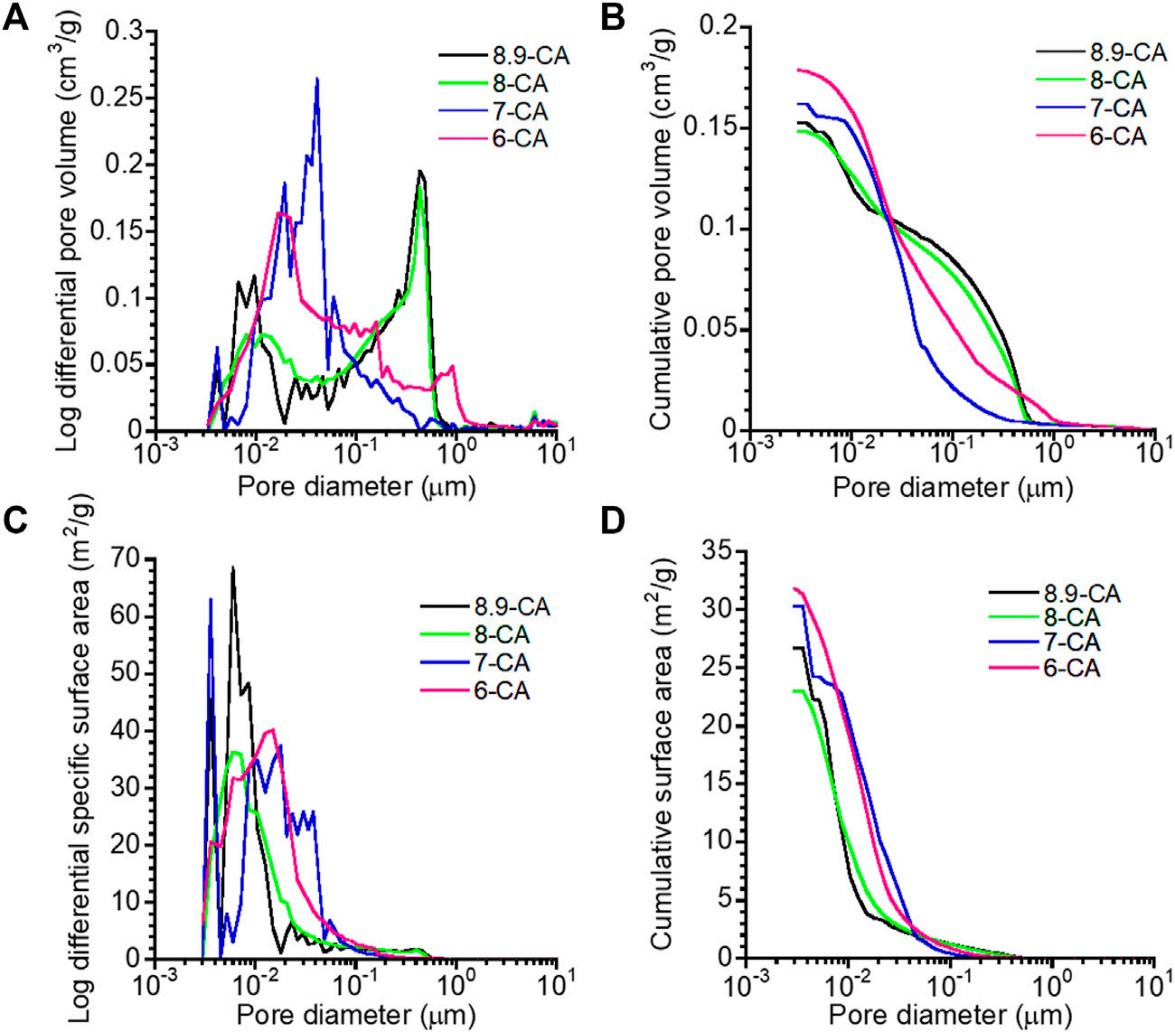
Open-pore distribution **(A)**, cumulative open-pore volume **(B)**, pore surface area distribution **(C)**, and cumulative surface area **(D)** versus pore diameter of 6-CA, 7-CA, 8-CA, and 8.9-CA.

The open-pore size distribution (Figure 4A) and open-pore volume (Figure 4B) of 6-CA, 7-CA, 8-CA, and 8.9-CA were measured using MIP. The open-pore distributions of 8.9-CA and 8-CA were similar, they had pores much larger than 10^−1^ μm and few pores smaller than 10^−1^ μm. In contrast, 7-CA and 6-CA had few pores larger than 10^−1^ μm and many pores smaller than 10^−1^ μm. Furthermore, the total open-pore volumes of 6-CA, 7-CA, 8-CA, and 8.9-CA were approximately 0.18, 0.16, 0.15, and 0.15 cm^3^ g^−1^, respectively. The pore surface area distribution (Figure 4C) and cumulative surface area (Figure 4D) of 6-CA, 7-CA, 8-CA, and 8.9-CA were calculated from the open-pore distributions. Pores smaller than 10^−1^ μm prominently contributed to high surface areas. The specific surface areas of 6-CA, 7-CA, 8-CA, and 8.9-CA were 31.8, 30.3, 23.0, and 26.7 m^2^ g^−1^, respectively (Table 2). Thus, the open-pore distribution and volume changed, reflecting the microarchitecture of the samples. This indicates that the pH of the phosphate solution used for the compositional transformation of CaCO_3_ to CAp can control the microarchitecture of the resultant CAp sample.

**TABLE 2:** Summary of MIP analysis.

Sample	Total open-pore volume/cm^3^ g^−1^	Specific surface area/m^2^ g^−1^
6-CA	0.18	31.8
7-CA	0.16	30.3
8-CA	0.15	23.0
8.9-CA	0.15	26.7

The open-pore volume and specific surface area were highest in 6-CA samples, indicating that the open-pore volume of the resultant CAp increased with decreasing pH. The 6-CA group was the only one in which no spherical aggregation was observed, suggesting that the plate-like aggregation structure composed of branched crystals affected the open-pore distribution and open-pore volume. These results suggested that, under weakly acidic conditions, CaCO_3_ dissolves quickly, resulting in microstructural alterations and, thus, a relatively high porosity. In general, open-pore volumes ≥0.15 cm^3^ g^−1^ promote osteoclastogenesis, contributing to bone maturation and bone formation within 4 weeks (Hayashi and Ishikawa, 2020). Porous surfaces with a specific surface area ≥19 m^2^ g^−1^ provide a suitable environment for cellular adhesion and proliferation (Meng et al., 2023), and accelerate the biodegradation of the materials (Diez-Escudero et al., 2017).

**FIGURE 5:**
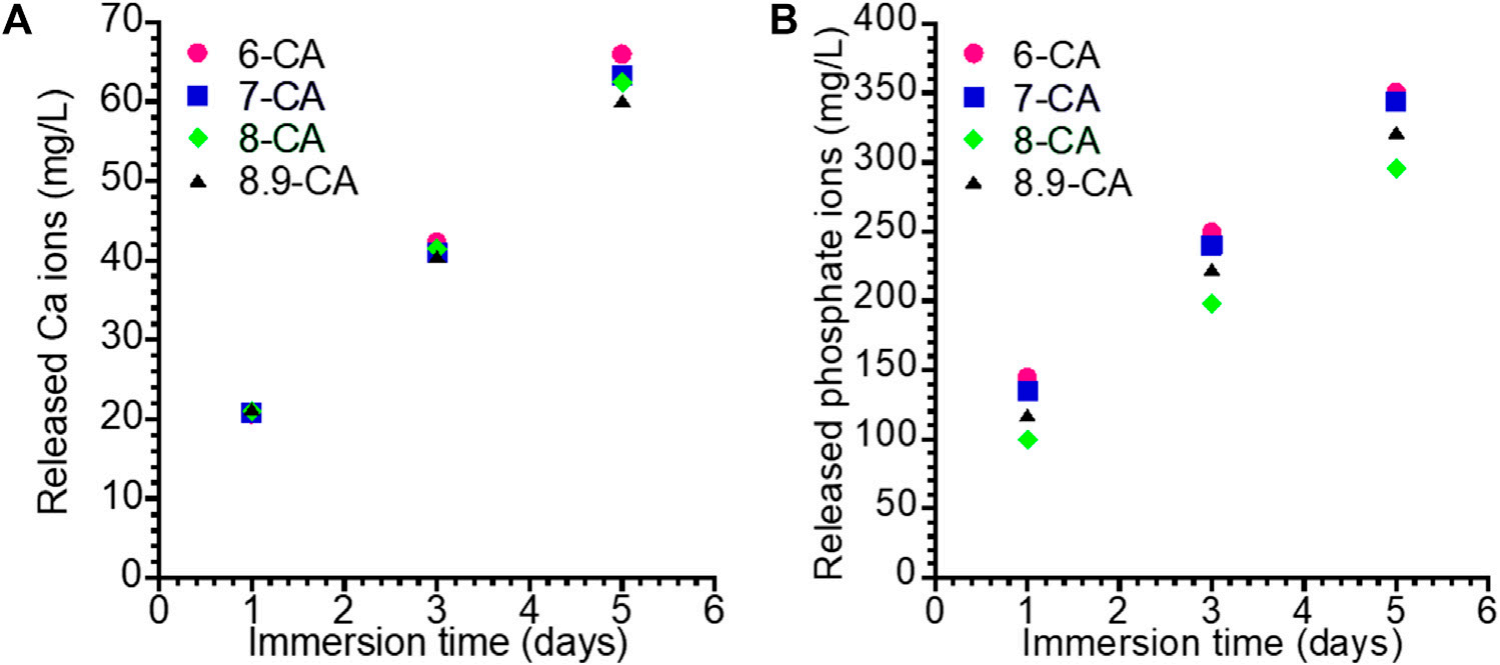
Concentration of the Ca **(A)** and phosphate **(B)** ions released form 6-CA, 7-CA, 8-CA, and 8.9-CA into the physiological saline.

For revealing the *in vivo* degradation behaviors of 6-CA, 7-CA, 8-CA, and 8.9-CA, these samples were immersed in physiological saline at 37°C for 1, 3, 5 days. The amount of Ca and phosphate ions released from each sample increased with time (Figure 5). The 6-CA exhibited the highest amounts of the released Ca and phosphate ions in all samples, indicating the 6-CA dissolved fastest *in vivo*. Moreover, the 7-CA showed higher amount of released Ca and phosphate ions than 8-CA and 8.9-CA. This suggests that CAp prepared at weakly acidic condition dissolves faster *in vivo* than that prepared at neutral and alkaline conditions.

Moreover, it is well known that osteoclastic resorption is faster for CAp containing larger amounts of carbonate in the apatite structure (Deguchi et al., 2022). Therefore, CAp prepared at weakly acidic condition may allow faster bone replacement based on the bone remodeling process. Histological evaluation is awaited based on the results obtained in this study.

## 4 Conclusion

Herein, we investigated the influence of the pH of a phosphate solution on the composition and architecture of CAp granules prepared from CaCO_3_ granules through a dissolution–precipitation reaction. The rate of compositional transformation from CaCO_3_ to CAp was accelerated under the pH 6.0 and pH 7.0 conditions. Moreover, the macroarchitecture of the resultant CAp granules did not change, even after the immersion of CaCO_3_ in a solution at pH 6.0 to 8.9, as they retained their granular shape. In contrast, the carbonate content of the resulting CAp granules varied depending on the pH. Moreover, the crystal morphology and microarchitecture of the resultant CAp granules were affected by pH under phosphate conditions. In particular, the open-pore distributions and volumes of the CAp granules prepared at pH 6.0–8.9 changed to reflect the microarchitecture of the samples. Therefore, the pH of the phosphate solution profoundly controlled the composition and microarchitecture of the resultant CAp granules prepared via the dissolution–precipitation reaction. Our findings provide fundamental insights into the design of CAp artificial bones to control bone formation based on composition and microarchitecture.

## Data Availability

The original contributions presented in the study are included in the article/supplementary material, further inquiries can be directed to the corresponding authors.
